# The Synergistic Effect of Electrical Stimulation and Dermal Fibroblast Cells-Laden 3D Conductive Hydrogel for Full-Thickness Wound Healing

**DOI:** 10.3390/ijms241411698

**Published:** 2023-07-20

**Authors:** Yen-Hong Lin, En-Wei Liu, Yun-Jhen Lin, Hooi Yee Ng, Jian-Jr Lee, Tuan-Ti Hsu

**Affiliations:** 1 x-Dimension Center for Medical Research and Translation, China Medical University Hospital, Taichung City 404332, Taiwan; 2 Department of Plastic and Reconstructive Surgery, China Medical University Hospital, Taichung City 404332, Taiwan; 3 School of Medicine, China Medical University, Taichung City 406040, Taiwan; 4 Department of Family Medicine, China Medical University Hospital, Taichung City 404332, Taiwan

**Keywords:** gelatin-methacrylate, graphene, cell-laden, electrical stimulation, wound healing

## Abstract

Clinically, most patients with poor wound healing suffer from generalized skin damage, usually accompanied by other complications, so developing therapeutic strategies for difficult wound healing has remained extremely challenging until now. Current studies have indicated that electrical stimulation (ES) to cutaneous lesions enhances skin regeneration by activating intracellular signaling cascades and secreting skin regeneration-related cytokine. In this study, we designed different concentrations of graphene in gelatin-methacrylate (GelMa) to form the conductive composite commonly used in wound healing because of its efficiency compared to other conductive thermo-elastic materials. The results demonstrated the successful addition of graphene to GelMa while retaining the original physicochemical properties of the GelMa bioink. In addition, the incorporation of graphene increased the interactions between these two biomaterials, leading to an increase in mechanical properties, improvement in the swelling ratio, and the regulation of degradation characteristics of the biocomposite scaffolds. Moreover, the scaffolds exhibited excellent electrical conductivity, increasing proliferation and wound healing-related growth factor secretion from human dermal fibroblasts. Overall, the HDF-laden 3D electroconductive GelMa/graphene-based hydrogels developed in this study are ideal biomaterials for skin regeneration applications in the future.

## 1. Introduction

Skin accounts for the largest surface area among all organs. It acts as the first-line barrier protecting the body from mechanical damage, ultraviolet radiation, thermal injury, and invasion by microorganisms. Irreversible and extensive cutaneous injuries may damage cutaneous integrity, which requires repair through a sequence of complex, coordinated, and active signal cascades of the wound healing process [1]. It comprises continuous layers of the epidermis, dermis, and subcutaneous tissues, which serve as protective barriers for the underlying muscular, vascular, and neural systems against harmful and toxic substances [2]. Cutaneous healing is a complex and dynamic process regulated by numerous intrinsic and extrinsic molecules divided into four successive but overlapping phases: Hemostasis, inflammation, proliferation, and remodeling [3]. The healing process often progresses in most superficial wounds; slight interruption at any stage may significantly impair wound closure, especially in large wounds or in patients with underlying diseases such as diabetes [4]. Therefore, the focus of recent research has now shifted to regenerative medicine, especially cutaneous wound healing and skin regeneration. With the growth in the aging population, there has been a corresponding increase in cutaneous injuries related to chronic diseases such as diabetes, which can lead to long-term complications [5]. The non-healing cutaneous injuries in 2020 alone cost approximately USD 55 billion, with thermal injuries costing an additional USD 8 billion. Despite improvements in medical knowledge and technology over the past decades, developments in the treatment for cutaneous injuries remain stagnant and plateaued, with most therapies being only moderately clinically effective, substantiating the need for effective therapies for cutaneous injuries. Therefore, the ideal scaffolds for skin regeneration should possess good biocompatibility, rich bioactivity, suitable biodegradation, and appropriate mechanical behaviors [6].

Hydrogels are an effective tool for cutaneous injuries because of their simple fabrication and capability to incorporate different types of biological molecules [7,8,9]. These incorporated molecules can be delivered with specificity and spatial control and are more effective than topical administration [10]. Furthermore, the properties and characteristics of hydrogels can be modified and tailored to suit different needs, such as anti-inflammatory, antioxidant, and antiseptic purposes [11,12]. Hydrogels are temporary skin replacements, offer protection, and enhance tissue regeneration [13]. In addition, the considerable water content in hydrogels provides an ideal environment for wound healing owing to their soothing and moisturizing properties [14,15]. Recent studies have emphasized the role of hydrogel-based bioinks crosslinked with three-dimensional bioprinting techniques in the treatment of fibrosis and the promotion of wound healing [16]. Gelatin has various properties and is widely used in tissue engineering and regeneration medicine [17,18]. Silk hydrogels modified by Chakraborty et al. using amine groups showed improved neovascularization and skin regeneration properties against third-degree burn wounds owing to enhanced biocompatibility and integration with host tissues [19]. Shi et al. developed a new process of cryogenic 3D biofabrication to manufacture scaffolds for skin tissue engineering [20]. This approach employed an extrusion free-form system and was found to be suitable post-treatments; moreover, the scaffolds exhibited excellent physicochemical behaviors and were fabricated to enhance biocompatibility. In addition, Guan et al. indicated that 3D-biofabricated hydrogel has beneficial effects on skin regeneration, exhibiting excellent printability and angiogenesis behaviors [21]. These studies show the potential of hydrogels for cutaneous lesions and emphasize the requirement for further improvements to enhance their efficacy for clinical applications [22,23].

Electroactivity can be incorporated into hydrogels to upregulate cellular activity and behavior. Conductive hydrogels are emerging as tools for wound healing. Several studies have shown that polyaniline, carbon nanotubes, and graphene exhibit excellent electroconductivity, improving wound healing in thicker granulation tissues [24,25,26]. However, these synthetic materials usually exhibit poor adhesiveness, restricting their application in skin regeneration [27]. Our previous study fabricated gelatin methacrylate (GelMa) with grooved topographies and poly(3,4-ethylenedioxythiophene):poly(styrenesulfonate) (PEDOT:PSS) [28]. The results demonstrated that the presence of PEDOT:PSS enhanced the mechanical behaviors of GelMa and further increased the swelling ratio of the hydrogels. It was recently reported that graphene-based composites had shown outstanding candidates for developing flexible, portable, and wearable electronics due to their superior mechanical properties and electrical conductivity [29]. Extrinsic electrostimulation guided the proliferation and migration of the seeded fibroblasts along the grooves of the scaffolds to attain a polar orientation. Other studies have shown that topical electrostimulation can decrease infection rates, enhance immunity, and increase perfusion, leading to efficient skin regeneration and wound healing [30]. In addition, graphene has also been shown to increase the proliferation of various cells [31]. It was found that graphene-containing scaffolds enhanced stem cell growth and maintained cells in an active proliferation state with the upregulation of Ki67 expression [32].

**Figure 1:**
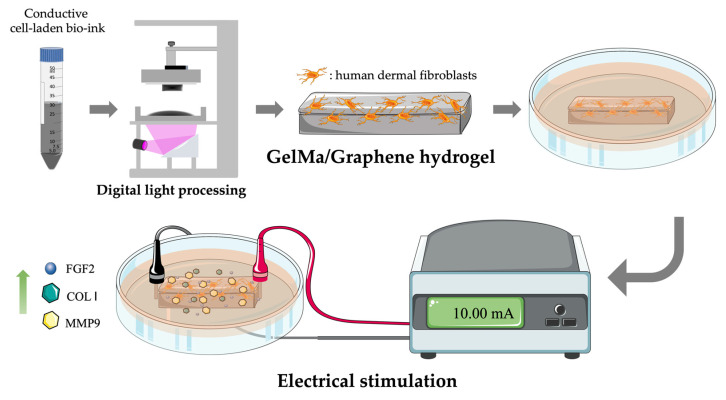
Schematic diagram of the GelMa/graphene scaffolds fabricated by DLP that considered the scaffolds with the ability to affect HDF proliferation by electrical stimulation and also promoted wound healing behaviors.

In this study, we mixed GelMA and graphene as biocomposites to synthesize conductive hydrogels and biofabricate cell-laden 3D scaffold with digital light processing (DLP) processing. Human dermal fibroblasts (HDF) were encapsulated in hydrogels, and electrical stimulation (ES) was applied (Figure 1). Our results demonstrated that the physicochemical properties, biodegradability, and cell behaviors were assessed to analyze the fidelity of the proposed composite hydrogels. Based on the above multiple performances, the HDF-laden graphene/GelMa scaffold further demonstrated a significant promotion. This composition may be a potential candidate for the clinical treatment of wound-healing applications or skin regeneration in the future.

## 2. Results and Discussion

### 2.1. Mechanical Properties of GelMa/ Graphene Hydrogel

**Figure 2:**
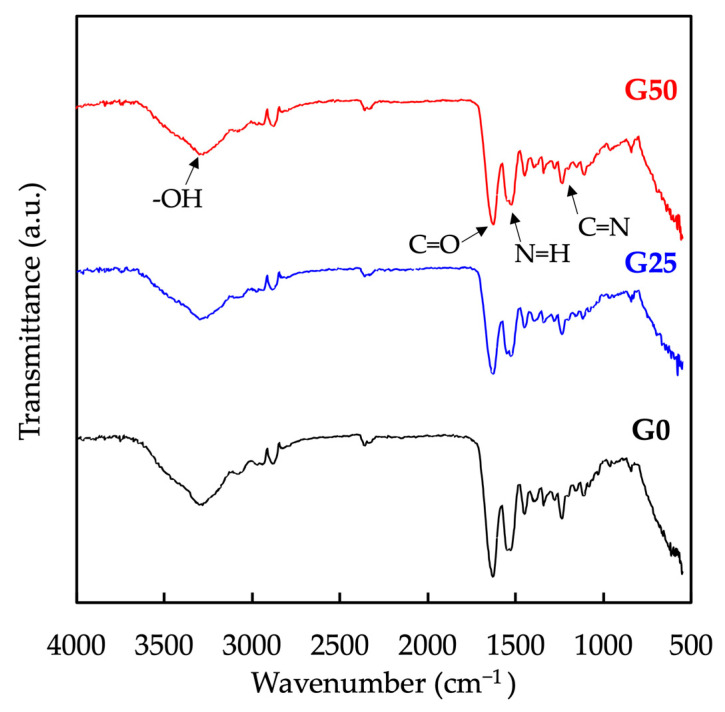
Fourier transform infrared spectroscopic (FTIR) spectrum of hydrogels for different concentrations of graphene.

FTIR spectra of the hydrogels were recorded to determine the components and functional groups of GelMa and graphene (Figure 2). The spectra of the samples consist of peaks at 3292 cm^−1^, 1630 cm^−1^, 1532 cm^−1^, and 1240 cm^−1^, which correspond to O-H, C=O, N-H, and C=N, respectively. These absorption bands are related to the functional groups of GelMa [33]. Compared to the spectrum of GelMa, the peaks of O-H and N-H were reported to be broadened in the spectrum of the GelMa/graphene hydrogel, which resulted from intermolecular interactions and hydrogen bonds between GelMa and graphene. Compared to the FTIR spectrum of the G0, the vibration peak of G50 shifted from 1630 to 1642 cm^−1^, which may be due to the strong van der Waals and π–π stacking interactions between GelMa and carbon-based material [34]. The FTIR spectra indicated successful crosslinking between the two materials. Furthermore, the addition of graphene did not significantly alter the structural composition of GelMa, thereby maintaining its individual properties.

**Figure 3:**
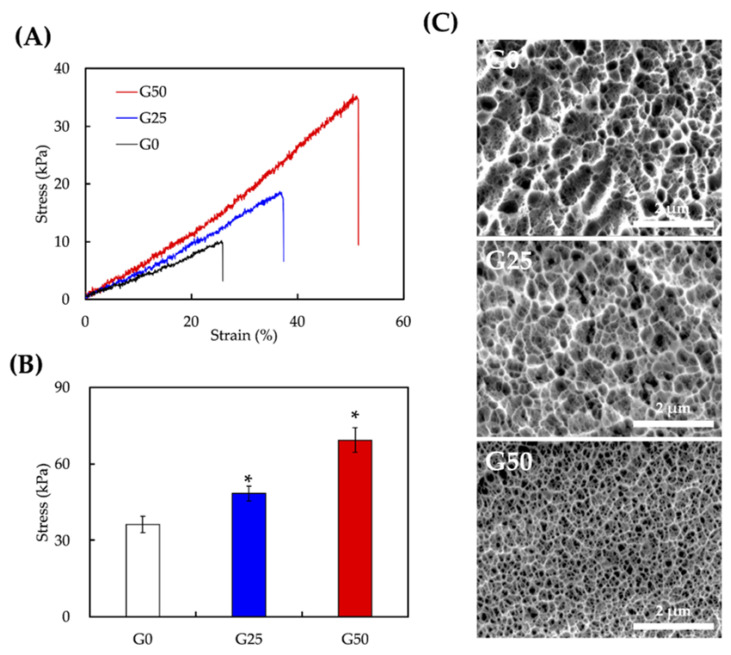
Mechanical properties of GelMA/graphene hydrogel. (**A**) Stress–strain curve, (**B**) tensile strength, and (**C**) hydrogel microstructure in G0, G25, and G50. Data presented as mean ± SEM, n = 6 for each group. * indicates a significant difference (*p* < 0.05) from G0.

The mechanical properties were evaluated using the tensile stress–strain test (Figure 3). The slope of the stress–strain curve showed that G50 had the highest stress at break compared to the other groups (Figure 3A). Moreover, it had the highest maximal tensile strength at approximately 69.23 ± 4.84 kPa among all groups, followed by G25 with 48.46 ± 2.76 kPa and G0 with 36.23 ± 3.23 kPa (Figure 3B). The mechanical properties were enhanced by the addition of graphene, which is associated with the intermolecular interactions and hydrogen bonding groups between graphene and the GelMa backbone [35]. Similar results have been observed in other studies, where the mechanical properties of composite hydrogels increased with the graphene concentration [36,37]. The increased elasticity and structural integrity provided sufficient mechanical strength for surgical implantation. The SEM images in Figure 3C show the surface morphology of the hydrogels. Increasing the concentration of graphene led to a higher density of the inner network with smaller pores, indicating that more crosslinks were formed between the graphene and GelMa.

**Figure 4:**
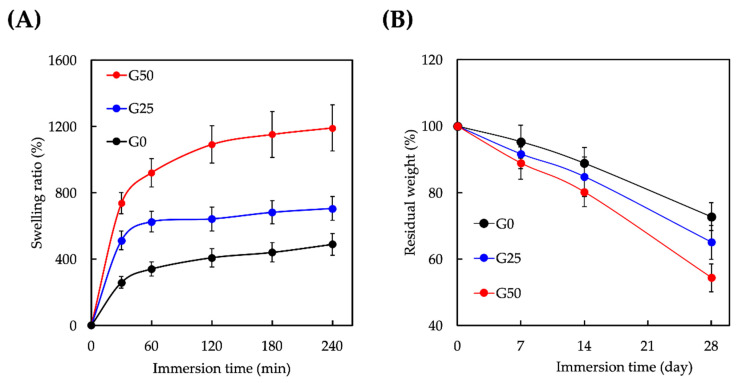
(**A**) Swelling ratio and (**B**) degradation rate of different GelMA/graphene hydrogels. Data presented as mean ± SEM, n = 6 for each group.

The effect of the graphene concentration on the swelling ratio and degradation rate of the scaffold was evaluated. As shown in Figure 4A, the percentage of volume expansion was assessed over 240 min. As expected, the swelling ratio was directly proportional to the graphene concentration. After 180 min of immersion, the swelling ratios of all the scaffolds gradually reached equilibrium. The results indicated that the graphene-contained GelMa led to increased cross-links between both materials, which improved the mechanical properties of the scaffold and enhanced integration between the surrounding tissues. To assess the percentage weight loss over 28 d, the residual weight of the scaffold was calculated (Figure 4B). Initially, the weight of the scaffold decreased steadily and dropped abruptly after 14 d of degradation. A greater degradation rate was observed with increasing graphene concentration, suggesting that adding graphene could accelerate the degradation of GelMa. As stated above, the GelMa-based hydrogel was reported to contain fewer amino acids than gelatin, which resulted in more heightened mechanical and fewer degradation behaviors suitable for wound healing [38].

**Figure 5:**
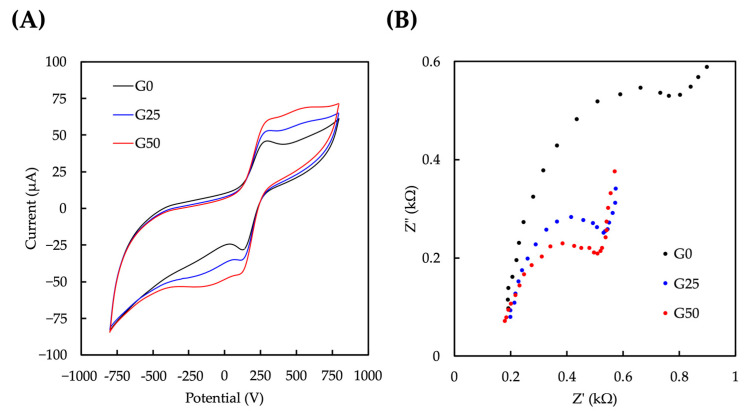
(**A**) The cyclic voltammogram and (**B**) Nyquist curves of different concentrations of GelMA/graphene hydrogel.

The previous study demonstrated that electrical stimulation could promote dermal fibroblast viability and promote cell migration and secretion of growth factors [39]. CV and EIS were performed to evaluate the conductivity and electrochemical behavior of the GelMa/graphene hydrogels. The CV plots for G0, G25, and G50 are shown in Figure 5A. The results indicate that the increase in the oxidative current is correlated with the graphene concentration, suggesting an enhanced electrical conductivity of the hydrogel due to the addition of graphene. According to the EIS profile, the electrical impedance decreased as the graphene concentration increased. This behavior indicated that graphene provided enhanced electrical conductivity and decreased the resistance of electrical signal transmission to the hydrogel [40].

### 2.2. Cell Viability and Proliferation

**Figure 6:**
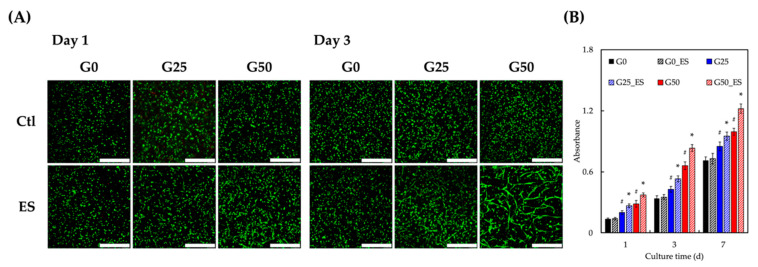
(**A**) Live/dead assay and (**B**) proliferation rate of HDF cultured in different concentrations of GelMA/graphene hydrogel with electrical stimulation. Data presented as mean ± SEM, n = 6 for each group. * indicates a significant difference (*p* < 0.05) from cultured without ES. # indicates a significant difference (*p* < 0.05) from cultured without G0. The scale bar is 300 µm.

The cellular viability and proliferation of HDF were encapsulated in the hydrogel, as well as the correlation between electrical stimulation and cellular behavior. Live and dead assays were conducted to evaluate the biocompatibility and cell viability at different concentrations of the graphene hydrogel (Figure 6A). Only a few dead cells (red) were observed in each group, suggesting good compatibility between the HDF, hydrogels, and electrical stimulation. Compared with day 1, cell proliferation increased in all groups after three days of culture, especially in those exposed to electrical stimulation. Thus, these results demonstrated the stimulatory effects of ES on HDF [41]. Moreover, similar to previous studies, a greater cell density was observed with an increased graphene concentration [42]. Among all groups, the cells in group G50 with ES had the highest cell density, with cell elongation, migration, and the creation of a polymeric network. In contrast, cells in the G0 and G25 phases, especially those without ES, tended to cluster and aggregate.

To evaluate the biocompatibility of the hydrogel and the effects of electrical stimulation, the cell proliferation rate was assessed using PrestoBlue (Figure 6B). After 3 and 7 days of culture, HDF in P4_ES had the highest proliferation rate among all groups, followed by those in G50, G25_ES, G25, G0_ES, and G0. In addition, the absorbance of the groups treated with electrical stimulation (G50_ES, G25_ES, and G0_ES) was higher than those not stimulated (G50, G25, and G0). These results demonstrate that electrical stimulation alone may contribute to the proliferation of HDF, indicating the absence of cytotoxicity in HDF. Furthermore, the proliferation rate was directly related to the graphene concentration, indicating a positive influence of electrical stimulation on HDF [41]. However, it is important to acknowledge that this study has certain limitations. The specific type of cell (HDF) in our experiments was used, and it is unclear whether the observed results would be consistent across different cell types. Further investigations involving other relevant cell types would be valuable to understand the generalizability of our findings.

### 2.3. Expression of Biomarker in Conductive HDF-Laden Hydrogel

**Figure 7:**
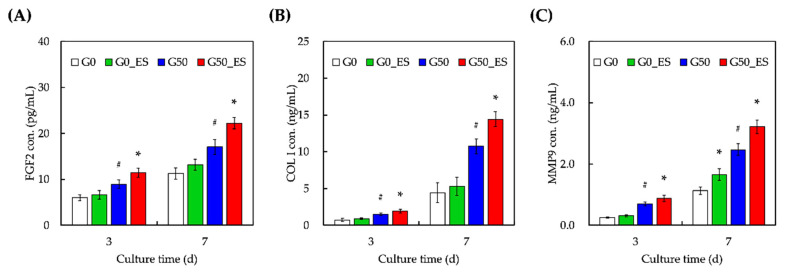
Expression of dermal regeneration-related growth factor, (**A**) FGF2, (**B**) Col 1, and (**C**) MMP9, expressed by HDF in different concentrations of GelMA/graphene hydrogel and treated with electrical stimulation (ES) after 3 and 7 days of culturing. * indicates a significant difference (*p* < 0.05) from G50. # indicates a significant difference (*p* < 0.05) from cultured without G0.

Dermal regeneration is affected by various growth factors, cytokines, and chemokines. To evaluate the feasibility of different hydrogels, several biomarkers involved in dermal regeneration were quantified (Figure 7), such as fibroblast growth factor 2 (FGF-2), collagen type I (COL 1), and matrix metalloproteinase (MMP9). FGF-2 promotes the growth, migration, and differentiation of dermal fibroblasts [43]. Similarly, type 1 collagen is an abundant component of the extracellular matrix of the skin that contributes to wound regeneration [44]. MMP9 participates in the regulation of ECM deposition and degradation during remodeling and re-epithelialization. Nevertheless, the timing of MMP activation and secretion is crucial for an ideal wound-healing process. Excessive MMP may lead to excessive matrix turnover, resulting in suboptimal wound healing [45]. As shown in Figure 7, all groups displayed increasing levels of all biomarkers after seven days of culture. ES contributed to the increased expression of FGF2, COL1, and MMP9 when comparing G0 and G0_ES [46]. Together, these results demonstrate that ES can enhance ECM remodeling and elevate better skin regeneration. The enlargement in the secretion of COL I and MMP9 was hypothesized to be due to the ability of ES and graphene to supply an improved mechanical, physicochemical, and cultured microenvironment for the HDF, directing to following downstream cellular behaviors.

### 2.4. In Vivo Rabbit Skin Wound Healing

**Figure 8:**
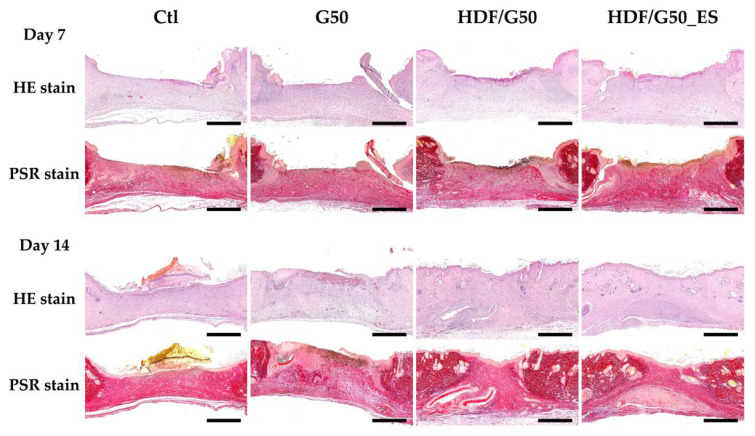
Hematoxylin-eosin (HE) and picrosirius red (PSR) staining used to evaluate new skin regeneration in the critical-sized defect in vivo 7 and 14 days after implantation. The scale bar is 250 µm.

The histological evaluation of in vivo wound healing was performed to observe the skin regenerative effects of the HDF, GelMA/graphene scaffolds, and ES. After 7 days and 14 days of scaffold implantation, the healing process of the critical-sized defect was assessed and presented in Figure 8. The HE and PSR staining was performed to evaluate the histological structure and collagen deposition (stain red) of the excised skin samples. Initially, the localized, 1 cm × 1 cm wound was created with the disruption of the entire epidermis and partial dermis. After 7 days of treatment, complete loss of epidermis and only little deposition of collagen were observed in the Ctl group, as shown in the HE stain and PSR stain, respectively. Compared to the Ctl group, the G50 group exhibited increased connective tissue in the dermis, while only incomplete epithelization was noted over wound edges. In addition, some neutrophil recruitment was noted, resulting in an inflammatory reaction in the healing wound. Additionally, the wound treated with HDF/G50 and HDF/G50-EF demonstrated enhanced dermal remodeling effects, such as increased collagen deposition and greater fibroblast density. Cell-laden hydrogel-based printed scaffolds might significantly enhance neovascularization and wound healing and are thus a great scaffolding candidate for skin tissue engineering [47]. Among G50, HDF/G50, and HDF/G50-EF, group G50 had the lowest levels of re-epithelialization with minimal epidermal growth, resulting in discontinued epidermis formation. The result indicated that the GelMA/graphene scaffold could serve as mechanical support during the cutaneous wound healing process, which provided structural stability and protection from external stimuli. Compared to all other groups, the scaffold with the addition of HDF showed increased recruitment of fibroblast, greater density of collagen deposition, and fewer inflammatory neutrophil recruitment, especially in the HDF/G50_ES group. Thus, thicker epithelium formation and uniformly distributed epithelium could be seen, indicating that HDF in our scaffold could expedite fibroblast migration during the wound healing process. After 14 days of treatment, reconstruction of the skin barrier could be seen in G50, HDF/G50, and HDF/G50_ES groups. Wounds in groups HDF/G50 and HDF/G50_ES were fully epithelialized with well-differentiated layers. Based on our findings, we concluded that incorporating HDF, electroconductive graphene, and electrical stimulation can lead to improved wound healing and enhanced skin regeneration. Our study focused on the histological evaluation of wound healing within a specific timeframe (7 and 14 days). Long-term effects and safety considerations were not assessed in this study. The GelMA/graphene scaffolds have the potential to interact with other organs in vivo, and it is crucial to investigate any potential effects or adverse reactions in future studies. Long-term safety assessments would provide a more comprehensive understanding of the biocompatibility and potential systemic impact of the GelMA/graphene scaffolds.

## 3. Materials and Methods

### 3.1. Synthesis of GelMa Bioink

Forty grams of gelatin (Sigma-Aldrich, St. Louis, MO, USA) was gradually added to a 360 mL phosphate buffer solution (PBS, Invitrogen, Grand Island, NY, USA) and was stirred and heated to 50 °C in distillation-distillation water (ddH_2_O) until fully dissolved. Next, 23 mL Methacrylic anhydride (MA) (Sigma-Aldrich) was slowly added to the mixed solution and left for 3 h until the reaction was complete. Next, the solution was diluted with 800 mL of warm ddH_2_O in the oven at 37 °C. Subsequently, the diluted solution was transferred to 50 mL centrifuge tubes and centrifuged at 8000 rpm for 5 min. Before the supernatant was poured into 10 K MWCO SnakeSkinTM dialysis tubing (Thermo Fisher Scientific, Waltham, MA, USA) and kept at 37 °C, care was taken that there were no suspended substances present. The ddH_2_O was changed, and the pH value of all samples was measured twice daily until dialysis was completed at a pH value of 6.1. Subsequently, the solution in the dialysis tubing was poured into a 1000 mL beaker and stirred evenly. Finally, the solution was transferred to plastic plates and kept in the −80 °C refrigerator for 1 d. All samples were freeze-dried until they became spongy solid specimens and stored at −20 °C for further use.

### 3.2. Cell Culture

The HDF (Cat: #2300, ScienCell Research Laboratories, Carlsbad, CA, USA) were cultured in a fibroblast medium (Cat: #2301, ScienCell Research Laboratories) supplemented with 2% fetal bovine serum, 1% fibroblast growth supplement, and 1% penicillin/streptomycin solution. The cells were incubated at 37 °C in a 5% CO_2_ environment, and the culture medium was changed every two to three days. Cells in passages 3–8 were employed to conduct the following experiments.

### 3.3. Preparation of Conductive Bio-Ink and Scaffold

To prepare the conductive bioink, a hydrogel was obtained by mixing specified amounts of 10% GelMa and 0.25% lithium phenyl-2,4,6-trimethylbenzoylphosphinate (LAP, Sigma-Aldrich) in PBS. Different concentrations of graphene (0%, 0.25%, and 0.5%) were added to the hydrogels and stirred evenly. The HDF was trypsinized, centrifuged, and added to the hydrogel at a density of 5 × 10^6^ cells/mL to obtain a conductive bio-hydrogel. The scaffold was drafted using SOLIDWORKS^®^ 2016 software. It was 2 mm in height and 8 mm in diameter. The film of the designed scaffold was then imported into the built-in software and printed using a 3D bioprinter (LumenX, Cellink, Gothenburg, Sweden). The thickness of each printed layer was set to 100 μm. The light-source intensity and total output power were set to 30 mW/cm and 30%, respectively. Each layer was irradiated for 3.5 s until the printing was complete.

### 3.4. Analysis of Scaffold Properties

The molecular functional groups and structural composition of the bioink were identified using Fourier transform infrared spectroscopy (FTIR) (Bruker, Ettlingen, Germany). The samples were printed with a diameter of 12 mm and a thickness of 2 mm. Then, the sample was analyzed via FTIR within a wavelength range of 4000 cm^−1^ to 500 cm^−1^ and a resolution of 1 cm^−1^. Tensile and mechanical properties were measured using a tabletop tensile tester (EZ-Test machine, Shimadzu, Kyoto, Japan). The mold and casting method was used to prepare a dumbbell-shaped tensile sample with a thickness of 2 mm. The samples were then stretched at a speed of 0.6 mm/min. The yield strength, strain, and Young’s modulus were recorded, and several graphs were generated based on the results. The averages and standard deviations were also calculated. At least eight specimens from each group were analyzed. A rheometer (MCR92, Anton Paar, Graz, Austria) was used to measure the rheological properties. Three hundred microliters of the bioink were placed in the rheometer, and the viscoelasticity changes while photo-curing under the UV light were analyzed at 37 °C. The intensity of the UV light was the same as the printing parameters.

### 3.5. Conductivity Measurement and Testing

An electrochemical analyzer simulator (ACIP100, Zensor R & D Co., Taichung City, Taiwan) was used to analyze the electrical conductivity of the scaffold. First, the electrode was coated with 50 µL bioinks uniformly. The forward and reverse potentials were scanned using cyclic voltammetry (CV) with a symmetrical sawtooth waveform, and the generated potentials were recorded. A test solution containing 5 mM red blood salt (K_3_[Fe(CN)_6_]) and 1 M KCl was used to create a cyclic voltammogram. The oxidation-reduction reaction of the electrodes was analyzed by scanning a redox probe at 100 mV/s in the range of −800 to 800 mV (relative to Ag/AgCl). In addition, the reactions of the samples under different frequencies and the impedance values were measured with electrochemical impedance spectroscopy (EIS). All experiments were conducted three times, and the average was calculated.

### 3.6. Electrical Stimulation

The samples were electrically stimulated once daily for 5 min at 10 mA, 100 μs, and 20 Hz using an electrical stimulator (Trio 300, Ito, Tokyo, Japan). An experiment was performed to evaluate the impact of electrical stimulation on cell behavior through the conductive bioink, and cellular viability and morphology were observed on days 3 and 7 of culture. All experiments were conducted three times, and the average was recorded.

### 3.7. Cell Proliferation

The scaffolds placed in the 48-well culture plate were kept at 37 °C in a 5% CO_2_ environment in the incubator, and the culture medium was changed every two to three days. Live/dead staining was performed to analyze the viability of HDF after 1, 3, and 7 d of culture. The HDF-laden scaffolds were rinsed thrice with PBS and incubated with a 1X LIVE/DEAD Viability/Cytotoxicity kit for 60 min. Following this, the live/dead cells were imaged using a fluorescence microscope. Live cells are labeled green, whereas dead cells are labeled red. All of the above processes were conducted in the dark. All experiments were conducted three times, and the average was recorded.

### 3.8. Quantification of Secreted Proteins

The levels of fibroblast growth factor-2 (FGF-2), collagen I (COL I), and matrix metallopeptidase 9 (MMP-9) in the culture medium were quantified using enzyme-linked immunosorbent assay (ELISA) kits, such as FGF-2 ELISA kit (ab246531, Abcam, Cambridge, MA, USA), COL I ELISA kit (MBS7607063, MyBioSource, San Diego, CA, USA), and MMP-9 ELISA kit (MMP-9, ab246539, Abcam). Specific ELISA kits for each protein were used according to the manufacturer’s instructions. Briefly, culture medium samples were collected at the end of the designated culture period. The supernatant was collected for analysis after centrifugation to remove cellular debris. ELISA kits from their respective suppliers were used to quantify the target proteins. The concentration of each protein was determined by measuring absorbance using a spectrophotometer. A standard curve was generated using the known concentrations of each protein provided in the kits. Three independent tests were conducted for each sample.

### 3.9. In Vivo Wound Healing

Healthy New Zealand rabbits (1.8–2 kg) were involved in vivo experiments, which were bred in the Experimental Animal Center of China Medical University. The experiments were conducted by the Animal Research Ethics Committee of the Laboratory Animal Center of China Medical University (CMUI-ACUC-2020-352). They were anesthetized with intramuscular injections of Zoletil (20 mg/kg) and Xylazine (10 mg/kg). Skin and dermis surgery of the wound defect was performed on the back of the animal to form a 1 cm × 1 cm defect region. Each defect group was categorized as follows: Ctl, G50, HDF/G50, HDF/G50 + ES, with “Ctl” denoting the absence of material coverage, “HDF” indicating the presence of G50 hydrogel with HDF, and “ES” representing the inclusion of electrical stimulation. Postoperatively, antibiotics (Carprofen at 5 mg/kg and Cefazolin at 20 mg/kg) were administered within three days. The rabbits were euthanized at 7 and 14 days after implantation, and skin samples were collected for paraffin embedding. Subsequently, each sample underwent staining with hematoxylin and eosin (HE) as well as Picro-Sirius Red (PSR) for histological analysis. The experiments were conducted in triplicate.

### 3.10. Statistical Analysis

One-way statistical analysis of variance (ANOVA) was used to analyze the significance of differences between different experimental groups in each experiment. Significant deviations in each sample were determined using Scheffé’s multiple comparison test. *p*-value < 0.05 was considered statistically significant, as indicated by “*” or “#” in the different group comparisons.

## 4. Conclusions

The design and fabrication of new conductive bioinks for wound healing remain a challenge owing to the regeneration bottleneck faced by the current biocomposites. Combining different biomaterials that take advantage of the tunable properties of conductive biomatrix could overcome these bottlenecks and lead to the development of a range of biomaterials that can be used in multiple applications. Our results demonstrated that we successfully admixed GelMa and graphene bioscaffolds while maintaining their structural properties. The physical behaviors of the composite scaffolds, such as the swelling ratio and tensile strength, were restrained using different concentrations of graphene. Similarly, the G50 hydrogel showed increased conductivity and biocompatibility compared to the G0. ES of G50 scaffolds promoted cellular proliferation in vitro. Daily ES processing was reported to be sufficient to increase cellular proliferation compared with scaffolds without ES. Finally, these results demonstrated an influential synergistic therapeutic approach for advancing wound healing through combining the cell-laden GelMa/graphene 3D scaffold with ES that will benefit clinical wound therapy in the future.

## Data Availability

Data are available in a publicly accessible repository.

## References

[1] Li R., Liu K., Huang X., Li D., Ding J., Liu B., Chen X. (2022). Bioactive Materials Promote Wound Healing through Modulation of Cell Behaviors. Adv. Sci..

[2] Salameh S., Tissot N., Cache K., Lima J., Suzuki I., Marinho P.A., Rielland M., Soeur J., Takeuchi S., Germain S. (2021). A Perfusable Vascularized Full-Thickness Skin Model for Potential Topical and Systemic Applications. Biofabrication.

[3] Zhang H., Ma W., Ma H., Qin C., Chen J., Wu C. (2022). Spindle-like Zinc Silicate Nanoparticles Accelerating Innervated and Vascularized Skin Burn Wound Healing. Adv. Healthc. Mater..

[4] Jiang Y.L., Wang Z.L., Fan Z.X., Wu M.J., Zhang Y., Ding W., Huang Y.Z., Xie H.-Q. (2022). Human Adipose-Derived Stem Cell-Loaded Small Intestinal Submucosa as a Bioactive Wound Dressing for the Treatment of Diabetic Wounds in Rats. Biomater. Adv..

[5] Levengood S.L., Erickson A.E., Chang F., Zhang M. (2017). Chitosan–Poly(Caprolactone) Nanofibers for Skin Repair. J. Mater. Chem. B.

[6] Xiao H., Chen X., Liu X., Wen G., Yu Y. (2023). Recent Advances in Decellularized Biomaterials for Wound Healing. Mater. Today Bio.

[7] Cao Y., Cong H., Yu B., Shen Y. (2023). A Review on the Synthesis and Development of Alginate Hydrogels for Wound Therapy. J. Mater. Chem. B.

[8] Chen Y.S., Ng H.Y., Chen Y.W., Cho D.Y., Ho C.C., Chen C.Y., Chiu S.C., Jhong Y.R., Shie M.Y. (2023). Additive Manufacturing of Schwann Cell-Laden Collagen/Alginate Nerve Guidance Conduits by Freeform Reversible Embedding Regulate Neurogenesis via Exosomes Secretion towards Peripheral Nerve Regeneration. Biomater. Adv..

[9] Shie M.Y., Fang H.Y., Kan K.W., Ho C.C., Tu C.Y., Lee P.C., Hsueh P.R., Chen C.H., Lee A.K.X., Tien N. (2023). Highly Mimetic Ex Vivo Lung-cancer Spheroid-based Physiological Model for Clinical Precision Therapeutics. Adv. Sci..

[10] Wang Y., Song P., Wu L., Su Z., Gui X., Gao C., Zhao H., Wang Y., Li Z., Cen Y. (2022). In Situ Photo-Crosslinked Adhesive Hydrogel Loaded with Mesenchymal Stem Cell-Derived Extracellular Vesicles Promotes Diabetic Wound Healing. J. Mater. Chem. B.

[11] Kim N., Lee H., Han G., Kang M., Park S., Kim D.E., Lee M., Kim M.J., Na Y., Oh S. (2023). 3D-printed Functional Hydrogel by DNA-induced Biomineralization for Accelerated Diabetic Wound Healing. Adv. Sci..

[12] Saha R., Patkar S., Pillai M.M., Tayalia P. (2023). Bilayered Skin Substitute Incorporating Rutin Nanoparticles for Antioxidant, Anti-Inflammatory, and Anti-Fibrotic Effect. Biomater. Adv..

[13] Chen Y.W., Wang K., Ho C.C., Kao C.T., Ng H.Y., Shie M.Y. (2020). Cyclic Tensile Stimulation Enrichment of Schwann Cell-Laden Auxetic Hydrogel Scaffolds towards Peripheral Nerve Tissue Engineering. Mater. Design.

[14] Ma Y., Wang Y., Chen D., Su T., Chang Q., Huang W., Lu F. (2023). 3D Bioprinting of a Gradient Stiffened Gelatin–Alginate Hydrogel with Adipose-Derived Stem Cells for Full-Thickness Skin Regeneration. J. Mater. Chem. B.

[15] Yeo M., Yoon J.W., Park G.T., Shin S.C., Song Y.C., Cheon Y.I., Lee B.J., Kim G.H., Kim J.H. (2023). Esophageal Wound Healing by Aligned Smooth Muscle Cell-Laden Nanofibrous Patch. Mater. Today Bio.

[16] Tan B., Gan S., Wang X., Liu W., Li X. (2021). Applications of 3D Bioprinting in Tissue Engineering: Advantages, Deficiencies, Improvements, and Future Perspectives. J. Mater. Chem. B.

[17] Wu J., Xiao J., Zhu M., Yang H., Liu J., Liu Y. (2023). Study of Physicochemical and Gelation Properties of Fish Gelatin from Different Sources. Appl. Sci..

[18] Chen Y.W., Lin Y.H., Lin T.L., Lee K.X., Yu M.H., Shie M.Y. (2023). 3D-Biofabricated Chondrocyte-Laden Decellularized Extracellular Matrix-Contained Gelatin Methacrylate Auxetic Scaffolds under Cyclic Tensile Stimulation for Cartilage Regeneration. Biofabrication.

[19] Chakraborty J., Mu X., Pramanick A., Kaplan D.L., Ghosh S. (2022). Recent Advances in Bioprinting Using Silk Protein-Based Bioinks. Biomaterials.

[20] Shi L., Hu Y., Ullah M.W., Ullah I., Ou H., Zhang W., Xiong L., Zhang X. (2019). Cryogenic Free-Form Extrusion Bioprinting of Decellularized Small Intestinal Submucosa for Potential Applications in Skin Tissue Engineering. Biofabrication.

[21] Guan G., Lv Q., Liu S., Jiang Z., Zhou C., Liao W. (2021). 3D-Bioprinted Peptide Coupling Patches for Wound Healing. Mater. Today Bio.

[22] Chen Y.W., Shen Y.F., Ho C.C., Yu J., Wu Y.H.A., Wang K., Shih C.T., Shie M.Y. (2018). Osteogenic and Angiogenic Potentials of the Cell-Laden Hydrogel/Mussel-Inspired Calcium Silicate Complex Hierarchical Porous Scaffold Fabricated by 3D Bioprinting. Mater. Sci. Eng. C.

[23] Lee A.K.X., Lin Y.H., Tsai C.H., Chang W.T., Lin T.L., Shie M.Y. (2021). Digital Light Processing Bioprinted Human Chondrocyte-Laden Poly (γ-Glutamic Acid)/Hyaluronic Acid Bio-Ink towards Cartilage Tissue Engineering. Biomedicines.

[24] Wu Y., Lu Y., Wu C., Chen J., Ning N., Yang Z., Guo Y., Zhang J., Hu X., Wang Y. (2021). Conductive Dual Hydrogen Bonding Hydrogels for the Electrical Stimulation of Infected Chronic Wounds. J. Mater. Chem. B.

[25] Yang T., Yang M., Xu C., Yang K., Su Y., Ye Y., Dou L., Yang Q., Ke W., Wang B. (2023). PEDOT:PSS Hydrogels with High Conductivity and Biocompatibility for in Situ Cell Sensing. J. Mater. Chem. B.

[26] Ting M.S., Vella J., Raos B.J., Narasimhan B.N., Svirskis D., Travas-Sejdic J., Malmström J. (2022). Conducting Polymer Hydrogels with Electrically-Tuneable Mechanical Properties as Dynamic Cell Culture Substrates. Biomater. Adv..

[27] Lin Y.H., Chuang T.Y., Chiang W.H., Chen I.W.P., Wang K., Shie M.Y., Chen Y.W. (2019). The Synergistic Effects of Graphene-Contained 3D-Printed Calcium Silicate/Poly-ε-Caprolactone Scaffolds Promote FGFR-Induced Osteogenic/Angiogenic Differentiation of Mesenchymal Stem Cells. Mater. Sci. Eng. C.

[28] Lee J.J., Ng H.Y., Lin Y.H., Liu E.W., Lin T.J., Chiu H.T., Ho X.R., Yang H.A., Shie M.Y. (2022). The 3D Printed Conductive Grooved Topography Hydrogel Combined with Electrical Stimulation for Synergistically Enhancing Wound Healing of Dermal Fibroblast Cells. Biomater. Adv..

[29] Panwar V., Babu A., Sharma A., Thomas J., Chopra V., Malik P., Rajput S., Mittal M., Guha R., Chattopadhyay N. (2021). Tunable, Conductive, Self-Healing, Adhesive and Injectable Hydrogels for Bioelectronics and Tissue Regeneration Applications. J. Mater. Chem. B.

[30] Zheng F., Li R., He Q., Koral K., Tao J., Fan L., Xiang R., Ma J., Wang N., Yin Y. (2020). The Electrostimulation and Scar Inhibition Effect of Chitosan/Oxidized Hydroxyethyl Cellulose/Reduced Graphene Oxide/Asiaticoside Liposome Based Hydrogel on Peripheral Nerve Regeneration in Vitro. Mater. Sci. Eng. C.

[31] Shie M.Y., Chiang W.H., Chen I.W., Liu W.Y., Chen Y.W. (2017). Synergistic Acceleration in the Osteogenic and Angiogenic Differentiation of Human Mesenchymal Stem Cells by Calcium Silicate–Graphene Composites. Mater. Sci. Eng. C.

[32] Jin Y., Zhang W., Zhang Y., Yang Y., Fang Z., Song J., Qian Y., Yuan W.-E. (2022). Multifunctional Biomimetic Hydrogel Based on Graphene Nanoparticles and Sodium Alginate for Peripheral Nerve Injury Therapy. Biomater. Adv..

[33] Sujan M.I., Sarkar S.D., Sultana S., Bushra L., Tareq R., Roy C.K., Azam M.S. (2020). Bi-Functional Silica Nanoparticles for Simultaneous Enhancement of Mechanical Strength and Swelling Capacity of Hydrogels. Rsc Adv..

[34] Li Y., Liu Y., Peng B., Li X., Fang T., Liu S., Liu J., Li B., Li F. (2022). Stretchable, Conductive, Breathable and Moisture-Sensitive e-Skin Based on CNTs/Graphene/GelMA Mat for Wound Monitoring. Biomater. Adv..

[35] Mendes A.X., do Nascimento A.T., Duchi S., Quigley A.F., Aguilar L.M.C., Dekiwadia C., Kapsa R.M.I., Silva S.M., Moulton S.E. (2022). The Impact of Electrical Stimulation Protocols on Neuronal Cell Survival and Proliferation Using Cell-Laden GelMA/Graphene Oxide Hydrogels. J. Mater. Chem. B.

[36] Huang Y., Li X., Poudel A.J., Zhang W., Xiao L. (2022). Hydrogel-Based Bioinks for 3D Bioprinting Articular Cartilage: A Comprehensive Review with Focus on Mechanical Reinforcement. Appl. Mater. Today.

[37] Zhang X., Zhang H., Zhang Y., Huangfu H., Yang Y., Qin Q., Zhang Y., Zhou Y. (2023). 3D Printed Reduced Graphene Oxide-GelMA Hybrid Hydrogel Scaffolds for Potential Neuralized Bone Regeneration. J. Mater. Chem. B.

[38] Lai W.Y., Lee T.H., Chen J.X., Ng H.Y., Huang T.H., Shie M.Y. (2021). Synergies of Human Umbilical Vein Endothelial Cell-Laden Calcium Silicate-Activated Gelatin Methacrylate for Accelerating 3D Human Dental Pulp Stem Cell Differentiation for Endodontic Regeneration. Polymers.

[39] Wang J., Tian L., Chen N., Ramakrishna S., Mo X. (2018). The Cellular Response of Nerve Cells on Poly-l-Lysine Coated PLGA-MWCNTs Aligned Nanofibers under Electrical Stimulation. Mater. Sci. Eng. C.

[40] Sharma A., Gupta S., Sampathkumar T.S., Verma R.S. (2022). Modified Graphene Oxide Nanoplates Reinforced 3D Printed Multifunctional Scaffold for Bone Tissue Engineering. Biomater. Adv..

[41] Jin L., Hu B., Li Z., Li J., Gao Y., Wang Z., Hao J. (2018). Synergistic Effects of Electrical Stimulation and Aligned Nanofibrous Microenvironment on Growth Behavior of Mesenchymal Stem Cells. Acs Appl. Mater. Inter..

[42] Han M., Yildiz E., Kaleli H.N., Karaz S., Eren G.O., Dogru-Yuksel I.B., Senses E., Şahin A., Nizamoglu S. (2022). Tissue-like Optoelectronic Neural Interface Enabled by PEDOT:PSS Hydrogel for Cardiac and Neural Stimulation. Adv. Healthc. Mater..

[43] Ying H., Zhou J., Wang M., Su D., Ma Q., Lv G., Chen J. (2019). In Situ Formed Collagen-Hyaluronic Acid Hydrogel as Biomimetic Dressing for Promoting Spontaneous Wound Healing. Mater. Sci. Eng. C Mater. Biol. Appl..

[44] Lin F.S., Lee J.J., Lee A.K.X., Ho C.C., Liu Y.T., Shie M.Y. (2020). Calcium Silicate-Activated Gelatin Methacrylate Hydrogel for Accelerating Human Dermal Fibroblast Proliferation and Differentiation. Polymers.

[45] Abalymov A., Parakhonskiy B., Skirtach A.G. (2020). Polymer- and Hybrid-Based Biomaterials for Interstitial, Connective, Vascular, Nerve, Visceral and Musculoskeletal Tissue Engineering. Polymers.

[46] Lu Y., Li H., Wang J., Yao M., Peng Y., Liu T., Li Z., Luo G., Deng J. (2021). Engineering Bacteria-Activated Multifunctionalized Hydrogel for Promoting Diabetic Wound Healing. Adv. Funct. Mater..

[47] Bashiri Z., Fomeshi M.R., Hamidabadi H.G., Jafari D., Alizadeh S., Bojnordi M.N., Orive G., Dolatshahi-Pirouz A., Zahiri M., Reis R.L. (2023). 3D-Printed Placental-Derived Bioinks for Skin Tissue Regeneration with Improved Angiogenesis and Wound Healing Properties. Mater. Today Bio..

